# Insights into Recent Advances of Biomaterials Based on Microbial Biomass and Natural Polymers for Sustainable Removal of Pharmaceuticals Residues

**DOI:** 10.3390/polym15132923

**Published:** 2023-07-01

**Authors:** Lăcrămioara Rusu, Elena-Mirela Suceveanu, Alexandra-Cristina Blaga, Florin Marian Nedeff, Daniela Șuteu

**Affiliations:** 1 Faculty of Engineering, “Vasile Alecsandri” University of Bacau, 157 Calea Mărăşeşti, 600115 Bacau, Romania; florin_nedeff@ub.ro; 2 Faculty of Chemical Engineering an Environmental Protection “Cristofor Simionescu”, “Gheorghe Asachi” Technical University from Iasi, 71 A Mangeron Blvd., 700050 Iasi, Romania; acblaga@tuiasi.ro

**Keywords:** biosorption, microbial biomass, natural polymers, pharmaceuticals, water treatment

## Abstract

Pharmaceuticals are acknowledged as emerging contaminants in water resources. The concentration of pharmaceutical compounds in the environment has increased due to the rapid development of the pharmaceutical industry, the increasing use of human and veterinary drugs, and the ineffectiveness of conventional technologies to remove pharmaceutical compounds from water. The application of biomaterials derived from renewable resources in emerging pollutant removal techniques constitutes a new research direction in the field. In this context, the article reviews the literature on pharmaceutical removal from water sources using microbial biomass and natural polymers in biosorption or biodegradation processes. Microorganisms, in their active or inactive form, natural polymers and biocomposites based on inorganic materials, as well as microbial biomass immobilized or encapsulated in polymer matrix, were analyzed in this work. The review examines the benefits, limitations, and drawbacks of employing these biomaterials, as well as the prospects for future research and industrial implementation. From these points of view, current trends in the field are clearly reviewed. Finally, this study demonstrated how biocomposites made of natural polymers and microbial biomass suggest a viable adsorbent biomaterial for reducing environmental pollution that is also efficient, inexpensive, and sustainable.

## 1. Introduction

The ecosystem and all forms of life on Earth are currently in peril due to the unregulated discharge of a variety of contaminants into water, air, or soil. The rapid industrialization and global population increase during the last century determined the release of a vast number of harmful substances into the environment. The majority of these organic and inorganic contaminants were found in surface water, groundwater, soil, and drinking water [1,2,3,4]. The removal methods of these pollutants have been extensively researched in recent decades, but they remains a hot topic in the global scientific community [2,4,5,6].

Water use has expanded dramatically over the last century, resulting in the depletion of natural water resources, the deterioration of fauna, and certain aspects of life quality. An estimated 4 billion people worldwide do not have or have limited access to safe drinking water, and millions die each year from debilitating diseases caused by contaminated water [2,7,8]. This issue, together with rising energy usage, has prompted the development of novel technology for producing drinking water with little energy consumption. As a result, one of the primary global issues today is the development of innovative, environmentally friendly, low-cost, and high-efficiency water treatment technologies [5,9,10]. The negative impacts of organic and inorganic pollutants (dyes, pharmaceuticals, toxic metals, metalloids, radionuclides, etc.) on ecosystems and their health dangers have been demonstrated over time, necessitating the use of increasingly complicated pollutant detection methods [4,6,11]. The release of pharmaceutical compounds, organic dyes, and heavy metal ions into the environment by companies such as the pharmaceutical, textile, pulp and paper, and food industries endangers human health and ecosystems [12,13,14].

Pharmaceuticals represent an important part of the non-biodegradable or hardly biodegradable compounds found in wastewater and wastewater treatment plant effluents (WWTP). They have been extensively studied in the last 15 years, both in terms of quantifying their presence in different environmental matrices, toxic effects (Table 1), and removal methods [15].

**Figure 1:**
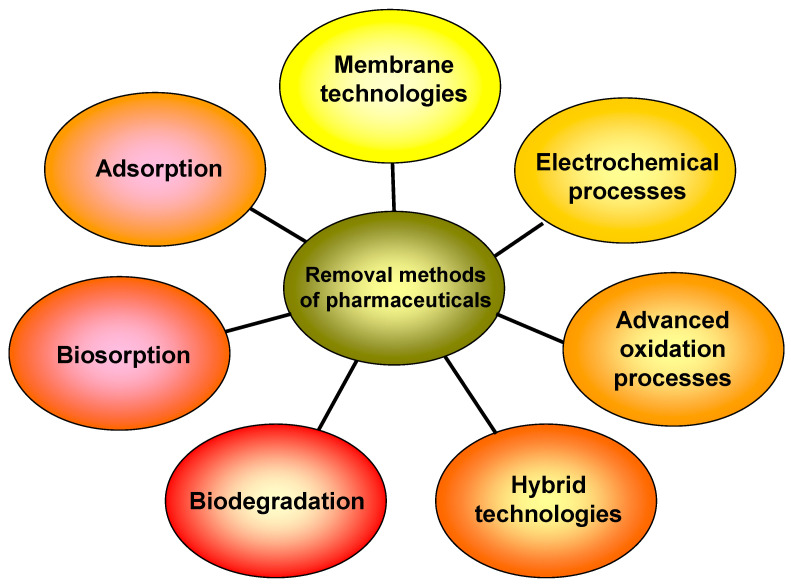
Various methods for removal of pharmaceuticals from aqueous matrices.

The global annual production of drugs has been estimated to be in the thousands of tons [2,6,21,22]. A wide range of methods (Figure 1), such as membrane separation, ozonation, flocculation, advanced oxidation, photocatalysis, microbial degradation, electrochemical processes, and adsorption, have been utilized to remove pharmaceuticals from aqueous matrices [3,7,8,23,24,25,26,27]. Most of these procedures involve the transfer of pollutants between different phases, the employment of additional chemicals, or the use of huge quantities of energy.

Furthermore, several of them generate trash and byproducts that must be treated in the following phases. Some of them, such as biological methods used in treatment plants, are ineffective at removing a variety of organic pollutants, such as contaminants of emerging concern, personal care products, pharmaceuticals, pesticides, endocrine disrupting compounds, dyes, and so on. These substances are stable and difficult to degrade, which has led to their accumulation in the environment [3,20,28].

**Figure 2:**
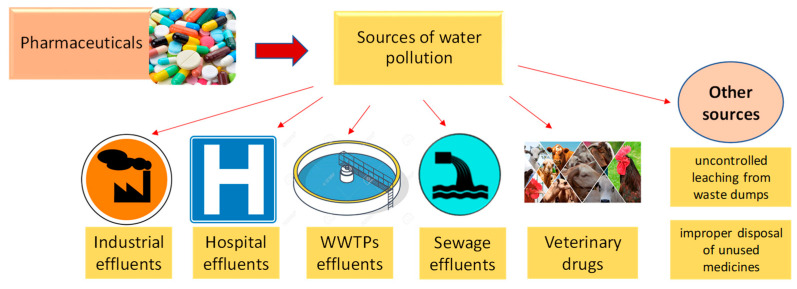
The main sources of water pollution with pharmaceuticals.

Pharmaceutical products and their transformation products can pollute surface water, underground water, and implicitly drinking water from a variety of sources (Figure 2), including wastewater treatment plant effluents, uncontrolled leaching from waste dumps, the pharmaceutical industry, hospitals, animal feed, improper disposal of unused medicines, etc. [2,6,21,22].

The wastewater treatment plant effluents are regarded as the primary source since, in most cases, these compounds are not eliminated and are detected in the treated water [6,21,29,30].

Researchers reviewed the occurrence and availability of pharmaceutical compounds in the environment, especially in various aqueous matrices [4,30,31,32,33,34]. Javaid Akhtar et al. [3] analyzed the presence of commonly detected pharmaceuticals in different water sources, such as hospital effluents, wastewater treatment plant influents, industrial effluents, river effluents, urban effluents, and surface water. It is evident from the examination of the data that a variety of pharmaceuticals are present, including antibiotics, anti-inflammatory drugs, lipid regulators, beta blockers, anticonvulsants, and contrast agents in concentrations ranging from 2.9 ng/L to 1.1 × 10^5^ ng/L.

Deblonde et al. [4] reported six classes of pharmaceutical compounds, including over 50 drugs, in WWTP influents and effluents ranging from 0.079 to 56.63 g/L. It has been demonstrated that pharmaceuticals are removed in WWTP in varying percentages, ranging from 1.4% for antiepileptics (trimethoprim) to 95.1% for antibiotics (tetracycline); however, it was found that for the majority of studied compounds, the rate of removal is lowered.

Vasilachi et al. [20] conducted a study on the environmental and health risks associated with emerging pollutants, in which they also made specific references to pharmaceutical compounds. The authors reported the toxicity of 35 residues of pharmaceutical compounds from 12 therapeutic groups in aquatic organisms and plants. The toxic effects of endocrine disruptors on human health were also presented, among which we mention: problems in the cardiovascular system, abnormal neural behaviors linked to obesity, altered endogenous steroid levels, etc., diabetes, and an altered reproductively relevant, sexually dimorphic neuroendocrine system.

Antibiotic-resistant bacterial strains have emerged as a result of persistent sublethal levels of antibiotic residues in aquatic environments [35]. The presence of these pollutants in natural water constitutes a serious risk, as stated by existing legislation [2,36], and several of them have already been designated as priority substances in water protection plans.

To assure the removal of pharmaceutical compounds from water, novel water treatment technologies must be developed. Recent research has concentrated on a number of techniques to accomplish this goal, but some of them come with the drawback that the breakdown of organic molecules can lead to new products with toxicity levels that are often even higher than the original chemicals [5,20,27].

Recent reviews mention the use of adsorption/biosorption processes for the removal of emergent contaminants from water and wastewater [10,11,21,37,38], but they are not focused on microbial biomass, its potential for drug removal, or the benefits and drawbacks of its use. Biosorption has emerged as a technology with considerable potential for the removal of these compounds from aqueous matrices in recent years, necessitating a linkage of information on biomaterials and the biosorption processes in which they might be used.

The purpose of this study is to provide an overview of current advances in pharmaceutical compound biosorption processes, with a special emphasis on microbial biomass and natural polymers as biosorbent materials, as well as the impact and obstacles connected with these investigations. This review also raises awareness about the importance of biomaterials in achieving a sustainable future. The biosorption potential of several biomaterials is explored, including microbial biomass, residual microbial biomass, natural polymers, biocomposites based on diverse inorganic compounds, and microbial biomass immobilized in natural polymers.

## 2. Bibliographic Research Methodology

The selection of studies for this review was completed using the Preferred Reporting Items for Systematic Reviews and Meta-Analyses (PRISMA) statement published in 2020 (with the checklists, explanation and elaboration, and flow diagram) [39].

The literature research strategy was developed in accordance with the PICO (Problem, Intervention, Comparison, Outcome(s)) framework, which is utilized to divide a topic into searchable components (Table 2).

The PICO strategy was used to conduct the research, and the following exclusion and inclusion criteria were established.

Inclusion criteria:

I1. Research articles published from 2010 to the present, full text;

I2. Removal of emerging pollutants—for automated screening, only the term “pollutant” was used;

I3. Removal of pharmaceutical compounds—for automated screening, only the term “pharmaceutical” was used;

I4. Evaluation of the application of microbial biomass (microbial cells, residual microbial biomass) and natural polymers for the removal of pharmaceutical compounds—manual screening;

I5. Relevance to the subject of the review (new information provided);

I6. Articles published or available in English.

Exclusion criteria:

E1. Articles published before 2010;

E2. Book or book chapters;

E3. Conference papers, notes, letters, short surveys, errata, or conference reviews;

E4. Articles published in languages other than English;

E5. Articles presenting the removal of pollutants monitored in routine studies (such as dyes and heavy metals, intensively studied pollutants).

**Figure 3:**
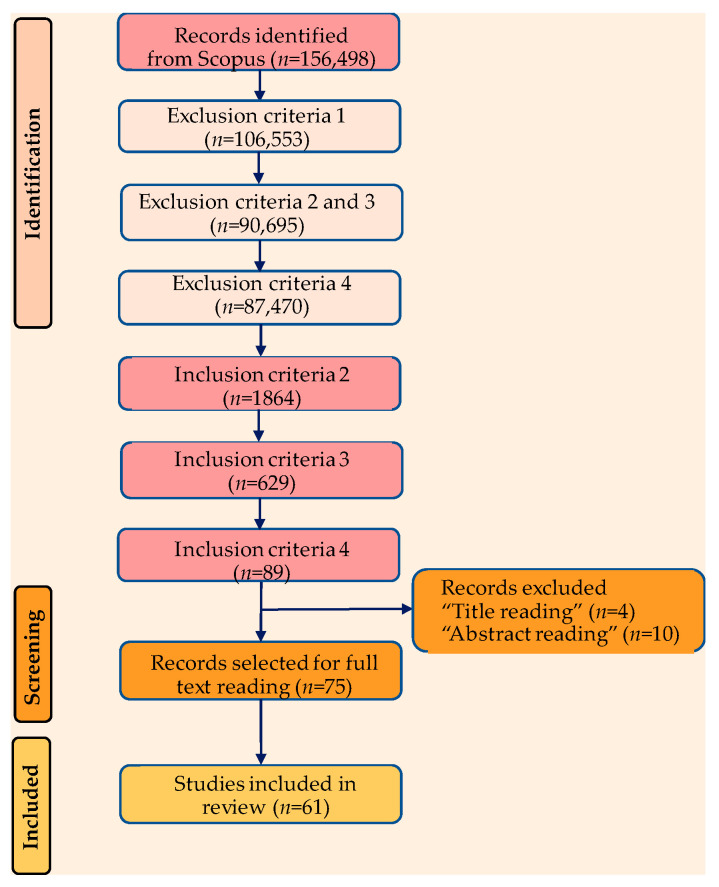
PRISMA flow chart of article selection process.

Using “biomaterial” as the primary search keyword, a literature search was conducted using the Scopus database (a comprehensive bibliographic database). Based on the aforementioned inclusion (I1 ÷ I3) and exclusion (E1 ÷ E4) criteria, papers were automatically chosen, and the decision to include them in the current review was made after carefully reading each manuscript (Figure 3).

A total of 61 articles covering the removal of pharmaceuticals using microbial biomass and natural polymers were chosen for inclusion in the present review after applying the full set of exclusion/inclusion criteria, including title, abstract, and full-text reading (Figure 3). Additional work has been added to the selected items in order to provide the required context. These articles were retrieved manually by using a “search and find” strategy and certain keywords (such as “biosorption”, “pharmaceutical removal”, etc.) in the Scopus database. Studies revealing the removal of dyes and heavy metals on the one hand and other biomaterials that are not based on microbial biomass and natural polymers on the other hand were not taken into consideration for inclusion in the current study.

The papers selected for this review present applications of microbial biomass and natural polymers for the removal of pharmaceutical compounds from aqueous matrices.

## 3. Biosorption—Concept and Current Perspectives

The remarkable capacity of microorganisms to remove organic and inorganic pollutants has been exploited in the development of biological methods for environmental depollution for a long time [11]. The concept of “biosorption” refers to a multifaceted process that depends on a number of factors, including the availability of various mechanisms, the type of bio-sorbent being employed, process parameters, and the presence or absence of metabolic activities in living organisms.

According to Fomina M. and Gadd G. M. [11], one of the significant properties of active and inactive microorganisms (and their components) is their ability to bind pollutants, which is utilized for the remediation of contaminants by biosorption and biodegradation. Their use, as well as the use of residual microbial biomass, microalgae, and agro-industrial waste in biosorption processes has produced impressive results for the removal of persistent, inorganic, and organic pollutants in low to medium concentrations in aqueous effluents [21,40,41,42].

Due to its ease of use (operation that is similar to that of traditional ion exchange technology), effectiveness in removing pollutants, and accessibility of biomass and residual biomass, biosorption has been regarded as a promising biotechnology for the removal and/or recovery of pollutants from aqueous solutions since the beginning of studies [11,21,41]. Great efforts have been made since the initial biosorption investigations to produce efficient, cheap, and adaptable biomaterials for wastewater treatment. Initially, studies were concentrated on the removal of metals and associated chemicals, but further biosorption research moved into additional areas of potential use, including pharmaceuticals [11,21,37,38,42]. Due to its dependence on the physical-chemical and biological properties of components (both pollutant and biosorbent), as well as the lack of clarity regarding the underlying mechanisms, biosorption is a complex process, as demonstrated by decades of research [11,41].

Most of the time, biosorption is considered a passive physico-chemical process, with mechanisms including: adsorption, ion exchange, and complexation. In reality, depending on the biosorbent-pollutant system and the given biosorption conditions, this can be an extremely complex process from the point of view of the mechanism [3,11]. In most cases, the term” biosorption” is assigned generically without regard to the mechanism, even if the process involves biodegradation or bioaccumulation.

Understanding the mechanism of interaction of target pharmaceutical compounds with microbial biomass-based biosorbents is mainly determined by the type of microbial cells involved, whether they are active or inactive. Thus, it can be said that in the case of active cells, the compound is removed by biodegradation and in the case of inactive cells by biosorption. Considering both the chemical structure of the pharmaceutical compounds in which different functional groups are present (i.e., phenolic, carboxylic, amide, amino, hydroxyl, alkoxide groups, etc.) or bonds (i.e., C=C aromatic) and the characteristics of the biosorbent, possible interactions that could describe the mechanism can be evaluated.

**Figure 4:**
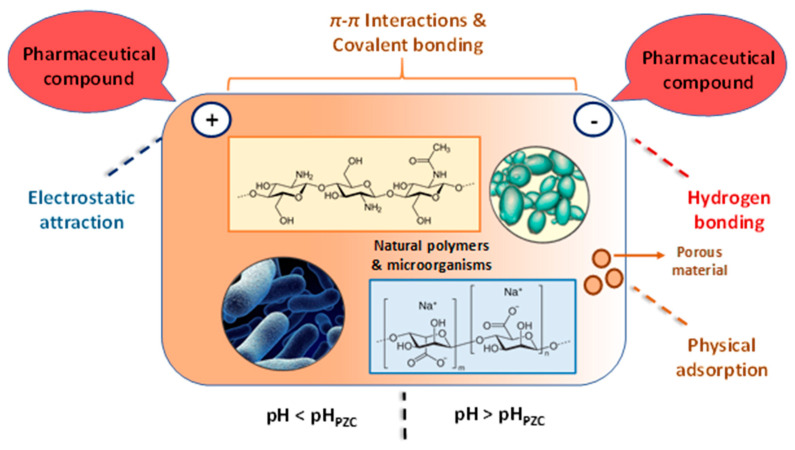
Schematic representation of the biosorption mechanism of pharmaceutical compounds (The figure was created taking into account the mechanism descriptions presented by Rashtbari et al. [43] (adapted with Elsevier permission, license 5578630226919, june 30, 2023); Grisales-Cifuentes et al. [44]; Samarghandi et al. [45]; Al-Gheethi et al. [46]; Yu et al. [47]; Akhtar et al. [3]; Rusu et al. [48]; Rusu et al. [49]).

As shown in Figure 4, four dominant mechanisms can be taken into account in the biosorption of pharmaceutical compounds on biosobents based on natural polymers and microbial biomass (inactive cells): (i) electrostatic attraction between the bio-sorbent and drug; (ii) π–π interaction between the surface of biosorbents and the pharmaceutical compound; (iii) hydrogen bonding interaction; (iiii) physical adsorption in the pores of the biosorbent. The pH value of the pollutant solution plays an important role in the biosorption process. Thus, at pH < pH_PZC,_ the electrostatic attraction is established between the negatively charged pharmaceutical compounds and the positive charge present on the surface of the biosorbent, and for pH values > pH_PZC_ the surface of the biosorbent has a negative charge, and H bonds (hydrogen bonds) can be made between the pharmaceutical compounds and it.

Samarghandi et al. [45] highlight the role of pH in the biosorption of amoxicillin (AMX) from aqueous solutions using *Saccharomyces cerevisiae*. The ionization of the substance in solution is influenced by pH, which also affects the adsorbent’s surface charge and biosorption capacity. The key components of AMX biosorption are functional groups on the biosorbent cell wall and active sites. As a result, the amines are proteinized, and their electrostatic charge changes to positive, allowing AMX to bind to the absorbent. The efficiency of AMX removal is significantly impacted by changes in the initial pH value. At a pH of 5, the removal efficiency was at its highest (81%).

The potential of living microorganisms to biodegrade pharmaceutical compounds underlies many of their removal processes.

**Figure 5:**
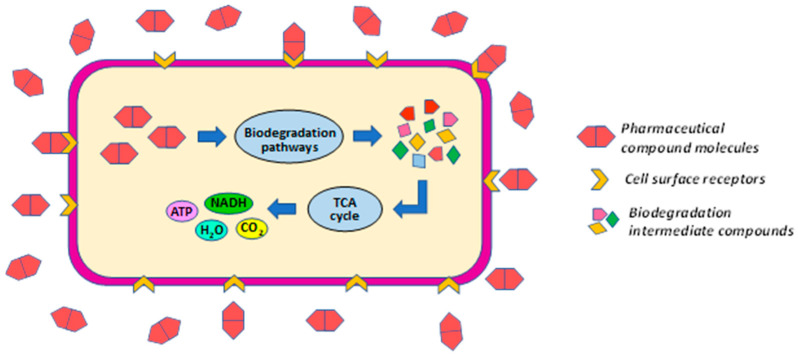
Predicted pharmaceutical compounds biodegradation mechanism. The biochemical degradation pathways represented here are created based on studies by Poddar et al. [16]; Adel et al. [50]; Wang et al. [51].

In the case of biodegradation, a primary degradation (Figure 5) can take place in which the pollutant in question is used as an energy source and is directly metabolized by the microbial strain to obtain energy. This type of degradation process takes place inside the cell, and the pollutant must be able to travel through the microbial cell wall to the cytoplasm. However, in many cases, due to the complexity of metabolic activities, complete metabolism of the pollutant is not achieved, and hazardous biodegradation products may result.

The *Pseudomonas* PrS10 strain of bacteria uses paracetamol as an energy source, according to an estimate of the biodegradation mechanism provided by Poddar et al. [16]. This is supported by a GC-MS study that reveals an insignificant presence of metabolites in the biodegradation broth.

Since studies so far have primarily concentrated on the removal of a single pollutant under static operating conditions and the use of biosorbents that are not viable for treating large volumes of water, biosorption has not yet achieved commercial success in its traditional direction as a low-cost and environmentally friendly depollution process [11,41]. Recently, there has been an increase in interest in the study of biosorption processes for binary or multiple solute systems, which are more typical of real-world wastewater and waste valorization problems [12]. The immobilization of biomass, which provides easy handling of biosorbents with good mechanical characteristics and easy separation, has markedly advanced the field of biosorbents domain [15,52].

In this context, it’s important to keep in mind the present trend of employing renewable bioresources to produce biosorbents [49,53]. On the other hand, both pragmatic market and cost justification should be considered when directing future research, so attention should be directed to alternative applications such as the recovery of pharmaceuticals, valuable metals, and elements, the manufacture of enriched feed and fertilizers, and the detoxification of food.

## 4. Biosorbents—Types and Applications in the Removal of Pharmaceutical Residues

Biosorbents are based on low-value products or wastes that are readily accessible in sufficient quantities, non-hazardous, cheap to acquire, and easy to use [11,12,37,40,41]. Analyzing the studies conducted so far (more than 13,000 scientific papers have been published in peer-reviewed journals to date), it appears that practically all biological materials have an affinity for organic and inorganic pollutants, indicating the enormous potential for biosorption [3,11,20,37,54,55].

As research has concentrated on identifying efficient and economical biosorbents as well as new opportunities for pollution control, a wide range of microbial and plant biomasses, as well as derived products, have been studied in various forms and in connection to various types of pollutants. As a result, the various biologically derived materials that have been extensively investigated to develop biosorbents include: microbial biomass (bacteria, cyanobacteria, filamentous fungi, yeasts, microalgae), marine algae (macroalgae), industrial waste (fermentation waste, food waste, activated sludge, sludge recovered from anaerobic fermentations, etc.), agricultural waste (fruit/vegetable waste, rice straw, wheat bran, sugar beet pulp, soybean husks, etc.), natural residues (residues of plants, sawdust, tree bark, weeds, peat), and other materials (chitosan, cellulose, seashell waste, etc.) [13,14,41,54,56,57,58].

Biosorbents as biological materials can be obtained using polymeric materials (alginate, chitosan, etc.) but also using microbial biomass, either free or immobilized. The microbial cells easily grown or available in large quantities in nature (bacteria, yeast, filamentous fungi) can be used in the pollutant’s removal process as active cells, when they are able to grow and reproduce in the polluted medium, but also in their inactive form, as dead cells, when the functional groups from the cell membrane or cell wall can still bind pollutants, but the cells do not need conditions compatible with life. In industrial activities (biotechnology: production of organic acids, amino acids, antibiotics, etc.), at the end of the biosynthetic process, the biomass is considered a by-product (industrial waste) that needs to be either valorized or disposed of. This is considered residual biomass (usually in inactive form) and can be used for the production of biosorbents. Most studies on pharmaceutical elimination use inactive microbial cells as biosorbents as the preferred approach to reduce complexity, as a microbial biosorbent that has been autoclaved performs better in biosorption as a result of the degradation of the cell wall, which leads to the appearance of additional binding sites. Active biosorbents have other advantages, as they could, through an active metabolism, modify the structure of the pollutant. Additionally, results on active biomass should not be disregarded because they have been utilized successfully to remove toxic metals and residues of persistent organic pollutants like dyes and pharmaceuticals [50,59].

The biosorptive capacities of different types of biomass have been reported in thousands of research papers and quantitatively compared in many reviews, from which it is evident that they can vary considerably and depend to a large extent on the experimental conditions and possible pretreatments applied [10,11,21,37,41,42,55,60]. The selection of the most promising biosorbents from a wide range of affordable and easily accessible biomaterials has been and continues to be the key problem. The goal is to obtain or choose biosorbents that are appropriate for industrial application for the greatest variety of large-scale persistent organic pollutants.

From this perspective, it could be based on (i) industrial waste (by-products from fermentative processes), which could be available for free or at a reduced cost; (ii) organisms that are readily obtainable in vast quantities in nature; and (iii) organisms that are easily cultivable.

### 4.1. Microorganisms and Residual Microbial Biomass

The use of microorganisms in biosorption processes began with the ability of biomass composed of active and inactive cells to form complexes with metal ions and was later expanded to include additional substances such as dyes and pharmaceutical contaminants. The inactive biomass has advantages such as low cost, low toxicity, ease of regeneration, ion exchange capacity, and adaptation to diverse pH and temperature values.

Biomass action is influenced not just by its chemical composition but also by external physico-chemical variables. Chelation, complexation, adsorption, ion exchange, degradation, electrostatic interaction, microprecipitation, coordination, and donor-acceptor interaction are frequently cited as mechanisms for micropollutant removal utilizing biomass [10,11,21,38,41]. Different types of microorganisms and residual microbial biomass have been used in the removal of pharmaceutical compounds by biosorption or biodegradation (Table 3).

Among the microorganisms used in biosorption/biodegradation processes are bacteria (Pseudomonas, Enterobacter, Streptomonas, Aeromonas, Acinetobacter, Klebsiella, Bacillus) and fungi (Myceliophthora thermophile, Trametes versicolor, Phanerochaete chrysosporium, Ganoderma lucidum), etc.

Numerous studies show that they can remove various pharmaceutical micropollutants from contaminated environments, e.g., ciprofloxacin, sulfamethoxazole, lomefloxacin, ofloxacin, norfloxacin, paracetamol, amoxicillin, sulfapyridine, sulfamethazine, diclofenac, ibuprofen, naproxen, iopromide, venflaxin, caffeine, and metoprolol [61,62,63,64,65,66,67].

**Table 3:** Various microorganisms and microbial biomass used for removal of several pharmaceuticals residues from aqueous matrices.

Biosorbent	Therapeutic Group/ Pharmaceutical Compound	Process Parameters	Obtained Results	Ref.
**BACTERIAL BIOMASS**				
	**Antibiotics**			
*Bacillus subtilis* 1156WTNCC strain (active cells)	Amoxicillin	pH = 6.5; temperature = 35 °C; time = 12 days; initial concentration of pharmaceutical compound = 0.2 ÷ 5.0 mg/mL; aerobic conditions; batch system	Maximum biodegradation efficiency 25.03% for C_0_ = 1 mg/mL	[68]
	Ampicillin		Maximum biodegradation efficiency 15.59% for C_0_ = 0.8 mg/mL	
	Cephalexin		Maximum biodegradation efficiency 22.59% for C_0_ = 1.0 mg/mL	
	Cefuroxime		Maximum biodegradation efficiency 10.62% for C_0_ = 1.0 mg/mL	
	Ciprofloxacin		Maximum biodegradation efficiency 2.45% for C_0_ = 0.6 mg/mL	
Bacterial community composed of *Desulfovibrio*, *Enterococcus* and *Peptostreeptococcus* spp. (active cells)	Ciprofloxacin	sulfate-reducing conditions; temperature = 25 ± 2 °C in the dark; time = 6 days; initial concentration of pharmaceutical compound = 1.0 mg/L; anaerobic conditions; batch system	Biodegradation efficiency was 85%, after 6 days	[64]
Bacterial community composed of *Comamonas*, *Arcobacter*, *Dysgonomonas, Macellibacteroides* and *Actinomyces*, genera (active cells)	Ciprofloxacin	nitrate-reducing conditions; temperature = 25 ± 2 °C in the dark; time = 6 days; initial concentration of pharmaceutical compound = 1.0 mg/L; anaerobic conditions; batch system	Biodegradation efficiency was 83%, after 6 days	[64]
*Acinetobacter* sp. (active cells)	Sulfamethoxazole	pH = 7.0; temperature = 25 °C in the dark; time = 6 days; initial concentration of pharmaceutical compound = 30.0 mg/L; in a shaker at 150 rpm; batch system	Biodegradation efficiency was 98.8%, after 10 h	[51]
*Bradyrhizobium* sp. GLC_01 strain (active cells)	Ciprofloxacin	biodegradation via cometabolism with another carbon substrate (glucose and sodium acetate); temperature = 25 °C; time = 8 days; initial concentration of pharmaceutical compound = 0.05 ÷ 10 mg/L; in a rotatory shaker at 150 rpm; batch system	Over 70% biodegradation was achieved at 0.05 mg/L whereas decreased to 26% at 10 mg/L	[69]
*Bacillus subtilis* strain (active cells)	Cephalexin	pH = 6.5; temperature = 35 °C; time = 12 days; initial concentration of pharmaceutical compound = 1.0 g/L; batch system	Biodegradation potential was 27, 22 and 21% in the presence of Ni^2+^, Cu^2+^, Zn^2+^ ions in solution at 10 mg/L concentration	[50]
Bacterial consortium composed of *Acinetobacter lwoffii* ACRH76, *Bacillus pumulis* C2A1, and *Acinetobacter* sp. HN_3_) (active cells immobilized or in suspension)	Ciprofloxacin	environmental conditions; temperature = 30 ± 2 °C; time = 20 days; initial concentration of pharmaceutical compound = 100.00 mg/L; floating treatment wetland strategy (FTWs)	Maximum biodegradation was 97% in the FTWs having immobilized bacteria	[70]
*Achromobacter* sp. JL9 with in-situ generated biogenic manganese oxides (active cells)	Sulfamethoxazole	pH = 7.0; temperature = 30 ± 1 °C; time = 84 h; initial concentration of pharmaceutical compound = 5.0 mg/L; in a shaker at 125 rpm; batch system	Maximum biodegradation was 97.43% for the Mn (II) concentration of 2 mg/L	[71]
Microbial community including *Proteobacteria, Bacteroidetes, Firmicutes, Actinobacteria* and *Armatimonadetes* (active cells)	Chlortetracycline	pH = 7.2; temperature = 5 ÷ 45 °C; time = 28 days; initial con-centration of pharmaceutical compound = 100 µg/L; aerobic conditions; in a shaker at 120 rpm; batch system	Biodegradation rates of 48.7% and 84.9% were achieved by acclimated microbial populations in one and four weeks, respectively for the initial chlortetracycline level of 100 µg/L	[72]
Mixed culture of heterotrophic bacteria from activated sludge from sewage treatment plants (active cells)	Sulfamethoxazole	pH = 7.0; initial concentration of pharmaceutical compound = 20 ÷ 50 µg/L; suspended growth reactor SGR with stirring at 400 rpm; 24-cycle biodegradation experiment in a single SGR; simultaneous removal of 5 drugs; aerobic conditions	Removal rates by biodegradation of 73.2 ± 21.3% were achieved by simultaneous removal of drugs	[73]
*Pseudomonas* sp. CE22 strain isolated from activated sludge (active cells)	Cephalexin	temperature =26 °C; time = 10 h; initial concentration of pharmaceutical compound = 10 mg/L; in a shaker at 200 rpm; batch system	Biodegradation over 90% after incubation for 10 h	[74]
Activated sludge bacteria (inactivated biomass)	Ofloxacin	pH = 7.0; temperature =25 °C; time = 48 h; initial concentration of pharmaceutical compounds = 100 ÷ 700 ng/mL; in an orbital shaker at 120 rpm; batch system	Removal efficiency 45% for C_0_ = 100 ng/mL and 21% for C_0_ = 700 ng/mL; maximum biosorbtion capacity 1.5 ± 0.03 mg/g TSS	[75]
	Norfloxacin		Removal efficiency 50% for C_0_ = 100 ng/mL and 39% for C_0_ = 700 ng/mL; maximum biosorbtion capacity 3.24 ± 0.05 mg/g TSS	
	Ciprofloxacin		Removal efficiency 59% for C_0_ = 100 ng/mL and 43% for C_0_ = 700 ng/mL; maximum biosorbtion capacity 3.39 ± 0.06 mg/g TSS	
Bacterial consortium of *Burkholderia cepacia, Chrysomonas luteola, Pseudomonas fluorescens, Bacillus subtilis, Bacillus megaterium, Bacillus sterothermophilus, Citrobacter freundii, Kluyvera* (active and inactive cells)	Cephalexin	pH = 6.0; temperature =25 °C; time = 90 min; initial concentration of pharmaceutical compound = 0.2 ÷ 5.0 mg/L; in an orbital shaker at 125 rpm; batch system	Maximum biosorption efficiency (94.73% vs. 92.98% for living and dead cells respectively) was recorded at C_0_ = 0.4 mg/L, while for C_0_ = 5 mg/L dead cells exhibited more efficiency compared with living cells (82.36% vs. 46.66% respectively)	[46]
	**Antipyretics and analgesics**			
*Pseudomonas* PrS10 strain (active cells)	Paracetamol	pH = 7.2 ÷ 7.4; temperature =30 °C; time = 4–7 days; initial concentration of pharmaceutical compound = 3 g/L; in an orbital shaker at 140 rpm; batch system	Maximum biodegradation efficiency 96.37%, with 4.8 g/L of carbohydrate added, after 7 days	[16]
	**Anti-inflammatories**			
Mixed culture of heterotrophic bacteria from activated sludge from sewage treatment plants (active cells)	Ibuprofen	pH = 7.0; initial concentration of pharmaceutical compound = 20 ÷ 50 µg/L; suspended growth reactor SGR with stirring at 400 rpm; 24-cycle biodegradation experiment in a single SGR; simultaneous removal of 5 drugs; aerobic conditions	Removal rates by biodeg-radation of 24.2 ± 14.6% were achieved by simultaneous removal of drugs	[73]
Nitrifying bacteria isolated from activated sludge (active cells)	Ibuprofen	pH at approximately 7.5–8.0 during the incubation period; temperature =25 °C; initial concentration of pharmaceutical compounds = 25 ÷ 200 µg/L; in an orbital shaker at 80 rpm; batch system	Complete biodegradation (100%) at the lower concentration levels (25–100 µg/L) in 24 h	[76]
	Ketoprofen		Complete biodegradation (100%) at the lower concentration levels (25–100 µg/L) in 150 h	
Activated sludge bacteria (active cells)	Ibuprofen	pH = 7.0; initial concentration of pharmaceutical compound = 50 ÷ 300 mg/L; temperature =30 °C; in a shaker at 100 rpm in the dark; aerobic conditions; batch system	Biodegradation to undetectable concentrations within 4 days for C_0_ = 250 mg/L	[77]
	Diclofenac		Biodegradation rate of 75% within 3 weeks for C_0_ = 300 mg/L	
*Brevibacterium* sp. D4 strain (active cells)	Diclofenac	temperature =25 °C; initial concentration of pharmaceutical compounds = 10 mg/L; time = 30 days; in an orbital shaker at 150 rpm; batch system	Biodegradation was 35% for C_0_ = 10 mg/L of drug as a sole carbon source; Periodic feeding with acetate as a supplementary carbon source increased biodegradation by up to 90%	[78]
	**Anti-epileptics**			
Mixed culture of heterotrophic bacteria from activated sludge from sewage treatment plants (active cells)	Carbamazepine	pH = 7.0; initial concentration of pharmaceutical compound = 20 ÷ 50 µg/L; suspended growth reactor SGR with stirring at 400 rpm; 24-cycle biodegradation experiment in a single SGR; simultaneous removal of 5 drugs; aerobic conditions	Removal rates by bio-degradation of 4.2 ± 2.3% were achieved by simultaneous removal of drugs	[73]
*Starkeya* sp. C11 strain and Rhizobium sp. C12 strain (active cells)	Carbamazepine	temperature =25 °C; initial concentration of pharmaceutical compounds = 10 mg/L; time = 30 days; in an orbital shaker at 150 rpm; batch system	Biodegradation was 30% for C_0_ = 10 mg/L of drug as a sole carbon source	[78]
	**Antidepressants**			
*Labrys portucalensis* F11 strain (active cells)	Fluoxetine (racemic mixture) and its enantiomers FLX	temperature = 25 °C; initial concentration of pharmaceutical compounds = 2 µM *÷* 21 µM; time = 56 days; in an orbital shaker at 130 rpm; protected from light; batch system	Complete biodegradation of both enantiomers at C_0_ = 2 µM for FLX as sole carbon source was achieved in 30 days; The enantiomers were partially degraded at initial concentrations of 4 and 9 µM. Complete biodegradation of the two enantiomers occurred in the presence of acetate as an additional carbon source at 4, 9, and 21 µM	[79]
	**Antiseptics**			
*Enterobacter hormaechei* ssp. *Xiangfangensis* KG_2_16S strain (active cells)	Basic fuchsine	temperature = 37 ± 2 °C; initial concentration of pharmaceutical compounds = 20 ÷ 100 mg/L; time = 72 h; in an orbital shaker at 100 rpm; batch system	Maximum biosorption capacity was 140.54 mg/g for C_0_ = 20 mg/L, with 4 g/L of sucrose added	[80]
	**Histamine-2 blockers**			
Mixed culture of heterotrophic bacteria from activated sludge from sewage treatment plants (active cells)	Ranitidine	pH = 7.0; initial concentration of pharmaceutical compound = 20 ÷ 50 µg/L; suspended growth reactor SGR with stirring at 400 rpm; 24-cycle biodegradation experiment in a single SGR; simultaneous removal of 5 drugs; aerobic conditions	Removal rates by biodegradation and biosorption of 60.8 ± 15.0% were achieved by simultaneous removal of drugs	[73]
	**Hormones**			
Bacterial community composed of *Comamonas*, *Arcobacter*, *Dysgonomonas*, *Macellibacteroides* and *Actinomyces*, genera (active cells)	17β-estradiol	nitrate-reducing conditions; temperature = 25 ± 2 °C in the dark; time = 6 days; initial concentration of pharmaceutical compounds = 1.0 mg/L; anaerobic conditions; batch system	Biodegradation efficiency was 84%, after 6 days	[64]
	Psycho-stimulants			
Mixed culture of heterotrophic bacteria from activated sludge from sewage treatment plants (active cells)	Caffeine	pH = 7.0; initial concentration of pharmaceutical compound = 20 ÷ 50 µg/L; suspended growth reactor SGR with stirring at 400 rpm; 24-cycle biodegradation experiment in a single SGR; simultaneous removal of 5 drugs; aerobic condition	Removal rates by biodegradation and biosorption of 5.3 ± 4.4% were achieved by simultaneous removal of drugs	[73]
**FUNGAL BIOMASS**				
	**Antibiotics**			
*Saccharomyces cerevisiae* (active cells)	Amoxicillin	pH = 2 ÷ 8, initial concentration of pharmaceutical compound = 5 ÷ 5 mg/L, the amount of biosorbent = 0.1 ÷ 1.5 g/L; contact time = 10 ÷ 240 min	The highest removal efficiency, 93%, was obtained for C_0_ = 5 mg/L, bioadsorbent dose 0.75 g/L, pH = 5, time = 120 min; the highest value of adsorption capacity was 12 mg/g in the same conditions	[45]
*Trametes versicolor* ATCC 42530 strain (active cells)	Sulfapyridine	pH = 4.5 ± 0.3; temperature = 25 °C; biomass dose 1.8 g/L (measured as dry weight); tank bioreactor with mechanical agitation at 115 rpm; 5 g/L of glucose added; aeration condition; batch model	Complete biodegradation (100%) for C_0_ = 21.4 ng/g within 26 days	[66]
	Sulfathiazole		Removal efficiency by biodegradation was 85.9% for C_0_ = 143.0 ng/g within 26 days	
	**Anti-inflammatories**			
*Trametes versicolor* ATCC 7731 strain (living cells and chemically inactivated cells)	Naproxen	pH = 4.0; temperature = 25 °C; initial concentration of pharmaceutical compounds = 50–100 μg/L; biomass dose 0.4 g/L (measured as freshly grown fungus culture); time = 24 h; rotary shaker at 70 rpm; batch model	Biodegradation efficiency was over 60% with living cells and biosorption efficiency was 14 ± 5% with inactivated cells	[81]
	Ibuprofen		Biodegradation efficiency was over 60% with living cells and biosorption efficiency was 32 ± 1% with inactivated cells	
*Trametes versicolor* Ganoderma lucidum (active cells)	Diclofenac	pH = 4.5; temperature = 25 °C; initial concentration of each pharmaceutical compounds = 50 μg/L; biomass dose 1 g/L (measured as dry weight); time = 7 days; shaken conditions (150 rpm); batch model	The overall removal (100%) of diclofenac and ibuprofen after 7 days of incubation were achieved by both strains, T. versicolor and G. lucidum, by abiotic, biosorption and biodegradation	[82]
	Ibuprofen			
*Trametes versicolor* *Irpex lacteus* *Trichoderma reesei* (active cells)	Diclofenac	pH = 5.5; temperature = 25 °C; initial concentration of each pharmaceutical compounds = 2.5 ÷ 5 mg/L; time = 3 h ÷ 14 days; shaking incubator (150 rpm); batch model and fungal biofilm	*T. versicolor* and *I. lacteus* was able to completely (>99.9%) remove diclofenac after 7 days, by both mechanisms: enzyme activity and biosorption	[83]
*Fusarium solani* *Pleurotus ostreatus* (active cells)	Diclofenac		*F. solani* indicated a maximum reduction of 90% of diclofenac after 21 days; *P. ostreatus* removed the diclofenac >99.9% after 14 days; The combination of *F. solani* and *P. ostreatus* showed >80% removal of diclofenac after 14 days	[83]
*Trametes versicolor* (active cells)	Ketoprofen		Only *T. versicolor* was able to reduce more than 80% of ketoprofen after 21 days of incubation	[83]
*Phanerochaete chrysosporium* (active cells)	Naproxen	pH = 3.2 ÷ 4.5; temperature = 30 °C; initial concentration of pharmaceutical compound = 1.0 mg/L; continuous aerating mode; time = 28 days; batch system	Removal efficiency was 80.55 ± 3.26 on day 7; A removal higher than 95% was achieved after the addition of 8.25% sodium hypochlorite for inhibiting contamination in the reactor, on day 21; More than 90% naproxen C_0_ = 10 mg/L) was removed by the crude enzyme in the first two days	[84]
*Ganoderma lucidum* (FP-58537-Sp strain) (active cells)	Diclofenac	pH = 4.5 ± 0.5; temperature = 25 °C; initial concentration of pharmaceutical compound = 47 ÷ 184 μg/L; time = 6–26 days; first batch system, orbital shaking (135 rpm), dark conditions	Total removal was 98 ± 15% of which 58 ± 8% by biodegradation and 40 ± 6%by biosorption	[63]
	**Anti-epileptics**			
*Trametes versicolor* *Ganoderma lucidum* (active cells)	Carbamazepine	pH = 4.5; temperature = 25 *°C*; initial concentration of each pharmaceutical compounds = 50 μg/L; biomass dose 1 g/L (measured as dry weight); time = 7 days; shaken conditions (150 rpm); batch model	Maximum removal was 32%, achieved by biosorption, using the combined fungal system	[82]
*Trametes versicolor* (active cells)	Carbamazepine	pH = 7.5; temperature = 34–37 °C; initial concentration of pharmaceutical compound = 1 ÷ 20 mg/L; time = 5 h–7 d; first batch operation, then a continuous mode	Around 80% was eliminated when the diluted synthetic medium was applied as feeding. An effective elimination was achieved in ~100 days continuous operation, if sufficient nutrients were supplied	[85]
*Phanerochaete chrysosporium* (active cells)	Carbamazepine	pH = 3.2 ÷ 4.5; temperature = 30 °C; initial concentration of pharmaceutical compound = 1.0 mg/L; time = 28 days; aerating mode; batch system	Removal efficiency was 32.55 ± 1.22% on day 7	[84]
*Stropharia rugosoannulata* (FBCC 475 strain) (active cells)	Carbamazepine	pH = 4.5 ± 0.5; temperature = 25 °C; initial concentration of pharmaceutical compound = 47 ÷ 184 μg/L; time = 6–26 days; first batch system, orbital shaking (135 rpm), dark conditions	Total removal was 86 ± 7%, of which 84 ± 7% by biodegradation and 2% by biosorption	[63]
*Ganoderma lucidum* (FP-58537-Sp strain) (active cells)	Carbamazepine		Total removal was 36 ± 7%, of which 31 ± 6% by biodegradation and 5 ± 1% by biosorption	[63]
	**Antidepressants**			
*Trametes versicolor* (ATCC #42,530 strain) (active cells)	Venlafaxine	pH = 4.5 ± 0.5; temperature = 25 °C; initial concentration of pharmaceutical compound = 47 ÷ 184 μg/L; time = 6–26 days; first batch system, orbital shaking (135 rpm), dark conditions	Total removal was 55 ± 8%, of which 53 ± 8% by biodegradation and 2% by biosorption	[63]
	**Lipid regulators**			
*Trametes versicolor* *Ganoderma lucidum* (active cells)	Gemfibrozil	pH = 4.5; temperature = 25 *°C*; initial concentration of each pharmaceutical compounds = 50 μg/L; biomass dose 1 g/L (measured as dry weight); time = 7 days; shaken conditions (150 rpm); batch model	Complete removal (100%) is attributed to high intracellular oxidative biological pathway	[82]
	Clofibric acid		Removal efficiency was 14%, achieved by biosorption with *T. versicolor* strain and 41% with both strains simultaneously	
	**Hormones**			
*Trametes versicolor* *Ganoderma lucidum* (active cells)	Progesterone	pH = 4.5; temperature = 25 *°C*; initial concentration of each pharmaceutical compounds = 50 μg/L; biomass dose 1 g/L (measured as dry weight); time = 7 days; shaken conditions (150 rpm); batch model	Total removal (100%) of progesterone was achieved by both strains, *T. versicolor* and *G. lucidum*, predominantly through biodegradation	[82]
	**Histamine-2 blockers**			
*Trametes versicolor* *Ganoderma lucidum* (active cells)	Ranitidine	pH = 4.5; temperature = 25 *°C*; initial concentration of each pharmaceutical compounds = 50 μg/L; biomass dose 1 g/L (measured as dry weight); time = 7 days; shaken conditions (150 rpm); batch model	Total removal (100%) of ranitidine was mainly attributed to biological removal of the live fungal biomass by intra- or extracellular oxidative mechanisms	[82]

The characteristics of bacterial cell walls (type, nature, and number of active sites; acidity and basicity; chemical composition; morphology, etc.) influence biosorption, with the main responsible mechanisms being extracellular ones, biodegradation through co-metabolism, biodegradation through substrate consumption, and adsorption [11,64,68,73,74,79]. In the case of fungi, the cell wall is responsible for these microorganisms’ ability to remove pharmaceutical contaminants. Their application is particularly widespread due to the enhanced availability afforded by the possibility of large-scale cultivation with high yields as well as the numerous genetic alterations they can undergo, among other things.

As can be observed from the information in Table 3, pH, temperature, biomass type and nature, the presence or absence of oxygen, the addition of an additional carbon source, light, and initial pollutant concentration are the primary variables that affect the biodegradation process.

The process is influenced by factors such as biomass dose, biomass type and nature, pH, ionic strength, and initial pollutant concentration. Because of the small size of the microbial cells, it is necessary to apply hydrostatic pressure in the treatment of polluted water, which might cause cell disintegration. However, when the biomass is immobilized using various techniques, very good results are obtained [45,63,66,81,83,86,87].

### 4.2. Biosorbents Based on Natural Polymers

Natural polymers have become increasingly used in wastewater treatment for pharmaceutical removal during the last few years, as can be seen from Table 4. Superior values are obtained for the removal of pharmaceuticals using alginate-based biosorbents compared with chitosan in the case of tetracycline and ibuprofen.

According to numerous studies on the subject, one of the key factors contributing to biopolymers’ perceived benefits in wastewater treatment is their potential to be both environmentally and economically sustainable [88]. Natural polymers are also renewable, sustainable, biodegradable, cost-effective, eco-friendly, non-toxic, biocompatible, and hydrophilic [89,90]. They also have a range of functional groups, such as hydroxyls (-OH) and amines (-NH_2_), to which contaminants might bind via chemisorption or physical adsorption during the water purification process [91].

**Table 4:** Various biosorbent based on natural polymers designed for removal of several pharmaceuticals residues from aqueous matrices.

Biosorbent	Therapeutic Group/ Pharmaceutical Compound	Process Parameters	Obtained Results	Ref.
**ALGINATE**				
	**Antibiotics**			
Alginate-graphene-ZIF67 aerogel (AG-ZIF) Alginate-graphene-Co aerogel (AG-Co)	Tetracycline	pH = 6.0; room temperature; time = 720 min; initial concentration of pharmaceutical compound = 100 mg/L; adsorbent dose = 1 g/L; shaker at 150 rpm; batch system	Maximum adsorption capacities for AG-ZIF and AG-Co were 456.62 and 105.49 mg/g, respectively	[92]
Ga-based metal-organic gel/sodium alginate composite beads	Chlortetracycline hydrochloride	pH = 4.0 ÷ 8.0; temperature = 25 °C; initial concentration of each pharmaceutical compound = 20 mg/L; adsorbent dose = 1.00 g/L (measured as dry weight); time = 72 h; shaken conditions (150 rpm); batch model; dark environment	Maximum adsorption capacity was 1085.19 mg/g	[93]
	Ciprofloxacin hydrochloride		Maximum adsorption capacity was 862.07 mg/g	[93]
Polyvinyl alcohol-copper alginate gel beads	Tetracycline	pH = 3 ÷ 11; temperature = 20–45 °C; initial concentration of each pharmaceutical compound = 20 mg/L; adsorbent dose = 0.2 ÷ 2 g/L, time = 24 h; shaker at 150 rpm; batch system	Removal efficiency was 97.8% and the maximum adsorption capacity was 231.431 mg/g, when the dose of adsorbent is 2 g/L and temperature = 45 °C	[94]
	**Anti-inflammatories**			
Alginate/ Carbon-based films	Diclofenac	pH = 3 ÷ 11; temperature = 30–68 °C; initial concentration of each pharmaceutical compound = 10 ÷ 50 mg/L; adsorbent dose = 0.25÷2.0 g/L, time = 6 h; batch system under stirring of 400 rpm	Maximum DCF adsorption of 29.9 mg/g was obtained at pH = 3 and 30 °C	[95]
Alginate/polypyrrole/ ZnFe_2_O_4_ beads	Ibuprofen	pH = 5 ÷ 7; initial concentration of the pharmaceutical compound = 50–350 mg/L; beads dosage = 0.2–1 g/L (dry weight); temperature = 25–55 °C; contact time = 3 h; orbital shaking 150 rpm; batch system	Maximum adsorption capacity was 108.2 mg/g, that increase with 12% under an external magnetic field (EMF); C_0_ = 350 mg/L; adsorbent dose = 0.2 g/L, pH = 7.0, temperature = 25 °C; stirring 100 rpm	[96]
	**Antipyretics and analgesics**			
Calcium alginate/activated hydrochar composite beads	Paracetamol	pH = 6.5; initial concentration of the pharmaceutical compound = 100–250 mg/L; temperature = 15–35 °C; time = 10 h; shaker 150 rpm; batch system	Maximum adsorption capacity was 165.94 mg/g, for C_0_ = 250 mg/L, after 4 h at temperature = 25 °C	[97]
Alginate/polypyrrole/ ZnFe_2_O_4_ beads	Paracetamol	pH = 5 ÷ 7; initial concentration of the pharmaceutical compound = 50–350 mg/L; beads dosage = 0.2–1 g/L (dry weight); temperature = 25–55 °C; contact time = 3 h; orbital shaking 150 rpm; batch system	Maximum adsorption capacity was 106.7 mg/g, that increase with 14% under an EMF; C_0_ = 350 mg/L; adsorbent dose 0.2 g/L, pH = 7.0, temperature = 25 °C; stirring 100 rpm	[96]
	**Biomarkers**			
Alginate-graphene composites	Rhodamine B	initial concentration of the pharmaceutical compound = 5 mg/L; contact time = 24 h; darkness and gentle stirring; batch system	Maximum adsorption capacity of 178 mg/g was achieved for reduced graphene oxide-based beads	[98]
	**Antiretroviral**			
Activated carbon encapsulated in sodium alginate	Tenofovir disoproxil fumarate	pH = 4, an initial TDF concentration = 0.1 mM; temperature = 25 °C; adsorbent dose = 1 g of wet beads to 10 mL of TDF solution, the beads were whirled at 350 rpm	Maximum removal efficiency by adsorption was 92.68% after a contact time of 120 min	[99]
**CHITOSAN**				
	**Antibiotics**			
Chitosan-alginate-bentonite composites	Tetracycline	pH = 5.5; temperature = 25–50 °C; initial concentration of each pharmaceutical compound = 10–550 mg/L; adsorbent dose = 1–4 g/L; time = 240 min; batch system under continuous agitation	Adsorption efficiency was 97.7% for C_0_ = 10 mg/L at 50 °C after 30 min	[100]
Chitosan	Rifampicin	pH = 6.7; temperature = 20–45 °C; initial concentration of each pharmaceutical compound = 20–200 mg/L; adsorbent dose = 0.5–10 g/L; time = 240 min; orbital shaker (100 rpm); batch technique	Maximum adsorption capacity was 66.91 mg/g for C_0_ = 30 mg/L and adsorbent dose = 1.5 g/L	[101]
	Streptomycin		Maximum adsorption capacity was 11.00 mg/g for C_0_ = 30 mg/L and adsorbent dose = 1.5 g/L	[101]
Chitosan-based magnetic composite	Tetracycline hydrochloride	pH = 4 ÷ 12; temperature = 15–35 °C; initial concentration of pharmaceutical compound = 20–200 mg/L; adsorbent dose = 0.5 g (dry weight); time = 12 h; shaking speed of 140 rpm; dark environment; batch system	Maximum adsorption capacity was 50.2 mg/g for C_0_ = 100 mg/L at pH = 10 and temperature = 25 °C	[102]
Metal and clay embedded cross-linked chitosan	Tetracycline	pH = 2 ÷ 12; temperature = 25–45 °C; initial concentration of pharmaceutical compound = 20 mg/L; adsorbent dose = 0.5 g (dry weight); time = 24 h; orbital shaker 140 rpm; dark environment; batch system	Maximum adsorption capacity was 104.17 mg/g using zirconium loaded chitosan modified by perlite (Zr/Cht/Pt) composite at pH = 4; temperature = 25 °C	[103]
CuCoFe_2_O_4_—Chitosan magnetic nanohybrid	Tetracycline	pH = 3.5 ÷ 11.5; temperature = 25–40 °C; initial concentration of pharmaceutical compound = 5–30 mg/L; adsorbent dose = 0.2–1 g/L; time = 30 min; batch system	Highest adsorption efficiency was 93.07% for C_0_ = 5 mg/L, pH = 3.5, contact time of 20 min, the dose of 0.4 g/ L, and temperature of 25 °C	[104]
Chitosan-curdlan composite magnetized by zinc ferrite	Tetracycline	pH = 1.0 ÷ 11.0; temperature = 10–65 °C; initial concentration of pharmaceutical compound = 20–160 mg/L; adsorbent dose = 0.25–0.85 g/L; time = 120 min; batch system	Maximum adsorption capacity was 371.42 mg/g at 55 °C, C_0_ = 160 mg/L, 0.65 g/L dosage of adsorbent and pH = 6	[105]
Chitosan-carbon black waste composite beads	Amoxicillin	pH = 6.5 ÷ 8.5; temperature = 22 °C; initial concentration of pharmaceutical compound = 25–50 mg/L; composite beads dose = 10–20 g/L; time = 24 h; batch system	Maximum adsorption capacity was 12 mg/g for C_0_ = 25 mg/L (in demineralized water) and 15 mg/g for C_0_ = 25 mg/L (in tap water); pH = 6.5 ÷ 7.5	[106]
Chitosan-carbon black waste composite beads	Tetracycline		Maximum adsorption capacity was 39 mg/g for C_0_ = 25 mg/L (in demineralized water) at pH = 7.5 ÷ 8.5	[106]
	**Anti-inflammatories**			
Chitosan	Ibuprofen	pH = 6.7; temperature = 20–45 °C; initial concentration of each pharmaceutical compound = 20–200 mg/L; adsorbent dose = 0.5–10 g/L; time = 240 min; orbital shaker (100 rpm); batch system	Maximum adsorption capacity was 24.21 mg/g for C_0_ = 30 mg/L and adsorbent dose = 1.5 g/L	[101]
Chitosan-based magnetic composite	Diclofenac sodium	pH = 4 ÷ 12; temperature = 15–35 °C; initial concentration of pharmaceutical compound = 20–200 mg/L; adsorbent dose = 0.5 g (dry weight); time = 12 h; shaking speed of 140 rpm; dark environment; batch system	Maximum adsorption capacity was 123 mg/g for C_0_ = 100 mg/L at pH = 6 and temperature = 25 °C	[102]
Chitosan/Zr-MOF (UiO-66) composite foams	Ketoprofen	pH = 2 ÷ 9; initial concentration of pharmaceutical compound = 5–50 mg/L; adsorbent dose = 0.2 g/L; time = 10 h; shaking speed of 180 rpm; batch system	Maximum adsorption capacity of 209.7 mg/g was achieved for C_0_ = 50 mg/L at pH 4	[107]
Magnetic Fe/Cu–alginate nanocomposite beads	Naproxen	pH = 5.0; temperature = 25 °C; initial concentration of each pharmaceutical compound = 25 mg/L; adsorbent dose = 25 mg/30 mL of drug solutions; mixed at 250 rpm for 10 min; batch system	Removal efficiency was 84% for C_0_ = 25 mg/L and adsorbent dose = 25 mg/50 mL	[108]
Magnetic Fe/Cu-chitosan nanocomposite beads	Diclofenac sodium		Removal efficiency was 92% for C_0_ = 25 mg/L and adsorbent dose = 25 mg/50 mL	[108]
	**Psycho-stimulants**			
Chitosan/activated carbon composite beads	Caffeine	natural pH; temperature = 25 °C; initial concentration of each pharmaceutical compound = 50–800 mg/L; adsorbent dose = 1 g/L; time = 48 h; orbital shaker at 150 rpm; batch system	Maximum adsorption capacity was 391.00 mg/g for C_0_ = 10 mg/L	[109]
	**Non-ergot dopamine agonists**			
Chitosan grafted with sulfonic acid	Pramipexole	pH = 10; temperature = 25 °C; initial concentration of the pharmaceutical compound = 0–500 mg/L; adsorbent dose = 1 g/L; orbital shaker at 160 rpm; time = 24 h; batch system	Maximum adsorption capacity was 339 mg/g for C_0_ = 10 mg/L in the presence of 20 mg/L humic acid	[110]
	**Hormones**			
Chitosan nanoparticles	Estrogen	pH = 7.3; estrogen initial concentration = 3.5 ÷ 11.5 mg/L; adsorbent dosage = 1.45 g/L; time = 300 min. and magnetic stirrer at 600 rpm; batch system	Removal efficiency was 92.50% for C_0_ = 5.7 mg/L and contact time of 220 min	[111]
**OTHER NATURAL POLYMERS**				
	**Anti-epileptics**			
Ball-Milled Silk Fibroin Films	Carbamazepine	pH = 2–12; temperature = 15–45 °C; initial concentration of the pharmaceutical compound = 250 μg/L; time = 180 min; agitation speed of 100 rpm; batch system	Removal efficiency was 53% and adsorption capacity = 281 μg/g at pH = 12	[112]
	**Antibiotics**			
Heterogeneous natural polymer-based on dialdehyde inulin and laccase	Ofloxacin	pH = 4.5; temperature = 40 °C; initial concentration of the pharmaceutical compound = 25 mM; adsorbent dose = 8.5 mg/mL of the immobilized laccase; time = 60 h; stirring; batch system	Removal efficiency by biodegradation was 63% after 60 h of incubation	[113]
	**Biomarkers**			
Gelatin/activated carbon composite	Rhodamine B	pH = 2–11; temperature = 30–60 °C; initial concentration of the pharmaceutical compound = 50–50 mg/L; adsorbent dose = 3 g/L; time = 42 h; agitation speed of 100 rpm; batch system	Maximum adsorption capacity was 256.41 mg/g for C_0_ = 5.7 mg/L; pH = 4; temperature = 30 °C; time = 27 h	[114,115]
Poly(lactic acid)/activated carbon	Rhodamine B	pH = 2–12; temperature = 30–60 °C; initial concentration of the pharmaceutical compound = 100 mg/L; adsorbent dose = 2 ÷ 10 g/L; time = 54 h; agitation speed of 100 rpm; batch system	Removal efficiency was 88.99%; a highest adsorption capacity was 149.57 mg/g at pH = 4 and temperature = 60 °C	[116]

Alginate is a linear and anionic polysaccharide obtained from brown seaweeds such as *Laminaria hyperborea*, *Macrocystis pyrifera*, and *Ascophyllum nodosum*. This biopolymer is composed of alternating blocks of 1,4-L-guluronic acid (G) and 1,4-D-mannuronic acid (M) units and comes in a variety of grades depending on the purity necessary for a certain application [117]. Although it can also be obtained from bacterial sources, the commercial product is acquired in the form of a salt, such as sodium alginate, from algae. Alginate is well known for its biodegradability, low toxicity, and chemical versatility, but its unique property of forming a stable gel in aqueous media by the addition of multivalent cations makes this biopolymer useful in cell immobilization [48,117]. Aside from these, the remarkable cross-linking ability should be emphasized as a vital attribute for creating composite materials.

Chitosan is an amino-polysaccharide formed by N-deacetylation of chitin, which results in the formation of amine groups (-NH_2_) from acetamide groups (-NHCOCH_3_) [118,119]. Due to its unique properties, such as polyelectrolyte properties, biocompatibility, hydrophilicity, adhesion properties, biodegradability, and recyclability, chitosan has gained a great deal of interest in a variety of biomedical, water treatment, cosmetics, and food sectors, as demonstrated in numerous papers [42,120,121]. Chitosan has been identified as a promising cationic adsorbent for the removal of anions, heavy metals, toxic organic dyes, aromatic compounds, and pharmaceutical residues in recent studies, as can be seen from Table 4 and more [122,123,124,125,126,127].

This polymer has numerous advantages, including biodegradability, biocompatibility, high reactivity, hydrophilicity, and nontoxicity [122]. Furthermore, chitosan has a linear polyamine structure with a number of free amine groups that can be crosslinked and modified [42,52]. However, chitosan has several drawbacks, including low water resistance, a small specific surface area, incomplete recovery after adsorption, poor mechanical and thermal properties, a high agglomeration tendency, and high acid solubility [42,128]. As a result, several physical and chemical modification/functionalization techniques, such as sulfonation, carboxymethylation, and amination, were used to improve the adsorption characteristics and selectivity to remove pharmaceutical compounds from water as well as other emerging pollutants [129,130,131,132].

### 4.3. Biosorbents Based on Microbial Biomass Immobilized in Natural Polymers

The use of inactive microorganisms for the biosorption of pharmaceuticals offers a promising approach for the removal of these compounds from wastewater or contaminated solutions. While inactive microorganisms can effectively bind pharmaceutical compounds, it is important to note that the specific biosorption capacity and efficiency depend on several factors, such as the type of microorganism, surface properties, composition of pharmaceutical pollutants, and operating conditions. In addition, it is important to consider the possible release of pharmaceuticals from the biosorbent after use and the proper disposal or treatment of the spent biosorbent to avoid environmental contamination. Inactive microorganisms are commonly used for biosorption to reduce the need for maintenance, growth, and potential contamination risks associated with living organisms. The biosorption process using microorganisms often requires optimization of various parameters, such as pH, temperature, biomass dosage, contact time, and mixing, to maximize the biosorption efficiency [60].

Although the use of microorganisms for biosorption offers several advantages, there are also some disadvantages to consider: low specificity (different microorganisms have different affinities and selectivities, requiring screening for each pharmaceutical compound in order to obtain an effective biosorbent); slow kinetics (binding of pharmaceutical compounds with complex structures to the surface of the microorganism or their diffusion into the microorganism may be slower compared with other adsorbents); insufficient mechanical stability; difficult biosorbent regeneration; difficult separation from the aqueous phase due to the small size of the microbial cells [133].

**Figure 6:**
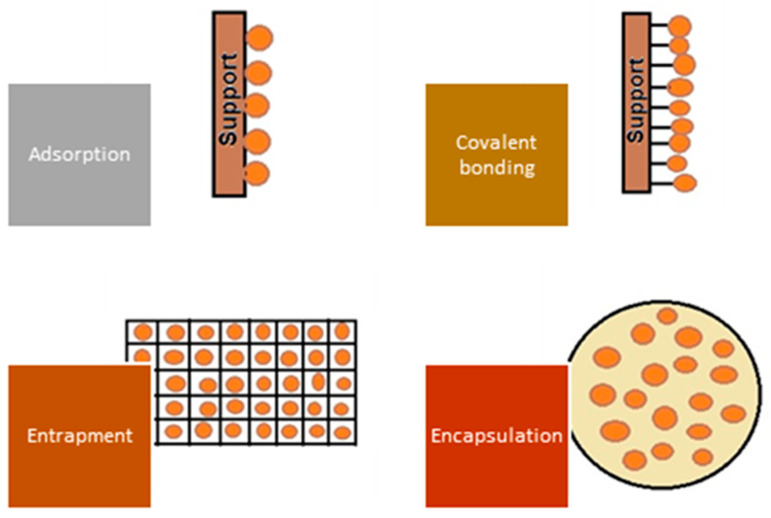
Representation of main immobilization techniques.

A significant improvement in the biosorption process’s performance is possible by using biosorbents obtained by immobilization (covalent bonding, cross-linking, encapsulation, entrapment in a polymeric matrix) of microorganisms or residual microbial biomass, as illustrated in Figure 6.

The entrapment consists of capturing cells inside gel-like materials forming capsules (beads with mechanical strength, stability, and durability but with mass transfer limitations (e.g., oxygen, substrate, or nutrients) with an internal structure similar to a network, protecting them from external threats and also from washout of the biomass without affecting its microbial capacity. The encapsulation of microorganisms (microencapsulation) is a kind of cell immobilization that consists of covering, coating, or trapping microorganisms in a very similar way to entrapment, with the difference of using a semi-permeable protective film, which allows for better nutrients and substrate on, variou transfer. Several microorganisms have shown high sorption capacities compared with conventional adsorbents (ion exchange resins and celluloses, activated carbon, and various polymeric materials). However, their use is limited due to physical problems that can be solved by immobilization to obtain particles with good physicochemical stability (higher mechanical strength) [134]. Several aspects need to be considered when choosing an appropriate immobilization technique, as immobilization involves additional costs, diffusion of the pollutant through the carrier can prolong the duration of the process and reduce its efficiency, and the biosorption capacity decreases due to the interaction between the support and the active functional groups of the biosorbent [15].

The term of immobilization refers to the process of binding or entrapping microorganisms in a matrix or support material so that they retain their activity and functionality and facilitate their use in various applications. The aim of immobilization is to produce 0.5 to 3.5 mm particles with good porosity, physical stability, and chemical resistance that can be used in dynamic systems (fixed bed or fluidized bed columns). There are several immobilization techniques that can be used for microorganisms and residual microbial biomass [135,136]:

– In entrapment, microorganisms are physically entrapped in a porous matrix or gel suitable for different types of cells. They are usually mixed with a gel-forming material such as calcium alginate, agar, or polyacrylamide, which solidifies into beads or capsules and immobilizes the microorganisms, allowing good mass transfer.
– In adsorption, microorganisms are bound to a solid support material by non-covalent interactions such as electrostatic forces, hydrogen bonds, or hydrophobic interactions. The support material can be in the form of particles, fibers, or a thin layer. Immobilization by adsorption is relatively easy, but the microorganisms may be desorbed over time.
– In covalent bonding, the microorganisms are chemically bound to the support material using cross-linking agents or functional groups. This method offers stronger and more stable bonding compared with adsorption but requires specific reactive groups on the microorganisms and the support material for successful bonding.
– In encapsulation, the microorganisms are enclosed in a semi-permeable membrane, or microcapsule. The membrane allows the diffusion of contaminants while protecting the microorganisms from external factors. Common encapsulation materials include polymers such as alginate, chitosan, or various gums. Encapsulation provides good protection for the microorganisms but can restrict mass transfer.

These immobilization techniques offer advantages such as improved stability, better reusability, and better control over the behavior of the microorganisms. The choice of immobilization technique depends on the specific application, the type of microorganism, the desired immobilization properties, and the conditions of use. Various biosorbents based on microbial biomass immobilized on natural polymers or other materials were analyzed for removing pharmaceutical residues from aqueous matrices (Table 5). The maximum biosorption capacity is evaluated under various operating conditions, and there is no standardized method for estimating the dry weight of the biomass and biosorbent used. As a result, studies from different authors can be difficult to compare.

The removal of pharmaceuticals from model, natural, and wastewater samples is obviously a capability of microbial biomasses, but the biosorption capacity of any biosorbent depends on its structure, pretreatment, chemical modification, immobilization technique, and also on the contaminant structure and properties. Analyzing the results obtained by Rusu et al., for the biosorption of cephalexin using the same microbial strain: Saccharomyces cerevisiae immobilized in calcium alginate [48] or chitosan [52], very different biosorption capacities were obtained for the two support materials: 93.34 mg/g compared with 22.78 mg/g, thus proving the importance of the polymer used for the immobilization. For the same biosorbent, Phanerochaete chrysosporium immobilized in wood chips, the removal efficiency varies significantly with the structure of the pharmaceutical compounds (Table 5).

Temperature usually increases the biosorption efficiency; pH controls the dissociation of both functional groups from the cell envelopes but also of the pharmaceuticals, providing binding sites. However, the support must be stable at the biosorption optimum conditions; chitosan, for example, is unstable (soluble) at a strong acidic pH. The microbial cells pretreatment methods also influence their efficiency, as through autoclaving or alkaline treatment, the cell wall is degraded and more functional groups are made available for biosorption. Initial pharmaceutical concentration can also have an impact on biosorption; at high concentrations, the amount of pollutant that is biosorbed per unit weight of the biosorbent increases, but removal effectiveness drops.

On the other hand, while the results obtained for free cells are occasionally better than those obtained for immobilized cells, the latter has the advantage of a high mechanical resistance, allowing the biosorbent to be used in a dynamic operating regime.

It is worth noting that the choice of the specific microorganism and the appropriate immobilization technique depends on factors such as the type and concentration of pharmaceuticals, the process conditions, and the objectives of the removal process. Further research and optimization are needed to determine the type of microbial biomass and the most suitable method of its immobilization to ensure efficient biosorption of pharmaceuticals under different conditions.

## 5. Conclusions and Future Perspectives

Pharmaceutical water pollution is a global issue that has an impact on both the environment and human health, especially when traditional approaches can’t provide an effective and secure solution; a large number of recent papers published underline the topic’s importance. According to this viewpoint, new environmentally friendly methods for removing pharmaceuticals from aqueous matrices must be developed, with the obvious goal of being applied at an industrial level.

The biosorption process can be efficient in removing pharmaceutical residues from various types of wastewater if a suitable biosorbent can be developed. From an ecological point of view, the use of biomaterials from renewable resources (microbial biomass, natural polymers) in biosorption processes has seen a constant increase in popularity in the last ten years.

This review evaluated the use of microbial biomass and natural polymers as biosorbents for the removal of pharmaceuticals from wastewater (simulated, real, or model solutions) by examining recent literature and taking into account the lack of in-depth analyses on this topic. The analyzed articles detailed several preparation and modification processes for biosorbents, demonstrating that microorganisms or natural polymers can provide extremely high biosorption capabilities and can be used to treat organically loaded wastewater in a more economic, efficient, and effective way than conventional wastewater treatment methods due to their high level of tolerance to contaminants. The production of efficient biosorbents (with improved adsorptive capacity and higher porosity) requires more analysis, as according to the literature data, natural polymers are less frequently utilized as single adsorbents and are more frequently used as composites, either with microbial biomass or with inorganic compounds. Therefore, research on microbial biomass or biopolymers is required to enhance the effectiveness of drug removal.

Future research should concentrate on the features of microbial biomass, whether it is active or inert, with the goal of achieving superior qualities and a good cost-benefit ratio acceptable for pharmaceutical removal. The use of microbial biomass in immobilized form could be helpful to future researchers and could serve as the basis for future advances, as it provides the framework for continuous system operation. These systems allow the treatment of large volumes of the liquid phase and should be applied for the study of real systems, i.e., wastewater from various polluted sources loaded with residues of pharmaceutical products. Additionally, it is crucial to optimize the water treatment process in pilot plants, for easy scale up. Biosorption studies focus on laboratory-scale applications, but they provide current knowledge of biosorption that is sufficient to provide a solid basis for expanding its use. The application of biosorption on an industrial scale has not yet been exploited, and this constitutes one of the major weaknesses that biosorption has to face.

Deeper studies covering biosorption on more emerging contaminants, including occur-ring mechanisms, more details on reaction intermediates and related pathways, related environmental aspects expressed in terms of water quality parameters, and more details on the effect of water parameters, are required in order to make biosorption an industrial process. Wherever feasible, biosorption should be incorporated into conventional techniques to create new secondary treatment systems to remove emerging contaminants, thus making these systems more profitable and environmentally friendly.

## Figures and Tables

**Table 1:** The toxic effects of some different therapeutic group of pharmaceuticals.

Therapeutic Group	Ecological and Human Health Effects	Reference
Antibiotics	Limit the therapeutic effectiveness of antibiotics used to treat human and animal infections by causing the development of antibiotic-resistant bacteria in the environment. Affects the growth of cyanobacteria and green algae, as well as leads to the development of antibiotic resistance in microorganisms. Acute and chronic exposure causes histopathological changes in some fish species	[5]
Analgesics	Increased production of antioxidant enzymes in mollusks (*Dreissena polymorpha*). Generate higher oxidative stress in fishes. Human exposure has been linked to serious health problems such as liver damage, myocardial infarction, nephrotoxicity, hypertension, cerebrovascular accidents, gastrointestinal bleeding, and fetal development impairment.	[16,17,18,19]
Anti-inflammatory	Can cause tissue damage to aquatic communities, affecting their growth and metabolism (i.e., alterations in gills and renal lesions at fish).	[5]
Endocrine disruptors	Human exposure can cause changes in the reproductively relevant, sexually dimorphic neuroendocrine system, alterations in endogenous steroid levels, diabetes, cardiovascular problems, aberrant neural behaviors, and is associated to obesity.	[20]

**Table 2:** PICO strategy applied in the present review.

**P** (Problem)	Presence of pharmaceutical compounds as emergent pollutants in different water sources
**I** (Intervention)	Applying innovative biomaterials based on microbial biomass and natural polymers to remove pharmaceuticals from aqueous matrices.
**C** (Comparison)	Biomaterials such as microbial cells, residual microbial biomass, natural polymers and biocomposites used in pharmaceutical compound removal processes
**O** (Outcome(s))	Improving pharmaceutical removal methods by identifying viable and sustainable materials to enable large-scale application of biosorption technologies.

**Table 5:** Various biosorbent based on microbial biomass immobilized on natural polymers or other materials designed for removing of several pharmaceuticals residues from aqueous matrices.

Biosorbent	Therapeutic Group/ Pharmaceutical Compound	Process Parameters	Obtained Results	Ref.
**ALGINATE—BASE FOR IMMOBILIZATION/ENCAPSULATION**				
	**Antibiotics**			
Saccharomyces cerevisiae/calcium alginate composite beads (inactive cells)	Cephalexin	pH = 2 ÷ 12; temperature = 25 °C; initial concentration of the pharmaceutical compound = 10–80 mg/L; biosorbent dose = 0.5 ÷ 3 g/L; time = 12 h; batch system	Removal efficiency was 86.23% for C_0_ = 30 mg/L; pH = 4; biosorbent dose = 1 g/L and the biosorption capacity was 94.34 mg/g	[48]
Saccharomyces pastorianus residual microbial biomass/calcium alginate composite beads Immobilization technique (inactive cells)	Cephalexin	pH = 5; initial concentration of the pharmaceutical compound = 10–80 mg/L; biosorbent dose = 0.5 ÷ 3 g/L; time = 12 h; room temperature; batch system	Biosorption capacity = 9.26 mg/g	[52]
	**Antiseptics**			
Saccharomyces cerevisiae/calcium alginate composite beads (inactive cells)	Ethacridine lactate	pH = 5; initial concentration of the pharmaceutical compound = 100 mg/L; biosorbent beads dose = 1 g/25 mL drug solution; time = 12 h; room temperature; batch system	Removal efficiency was 96.40%	[52]
Saccharomyces pastorianus/ calcium alginate composite beads (inactive cells)	Ethacridine lactate	pH = 2–10; initial concentration of the pharmaceutical compound = 20–60 mg/L; biosorbent beads dose = 1–3 g/L; time = 24 h; room temperature; batch system	Biosorption capacity was 26.72 mg/g and the removal efficiency was 91.05% for C_0_ = 60 mg/L; pH = 4; biosorbent dose = 2 g/L	[49]
Saccharomyces pastorianus residual microbial biomass/calcium alginate composite beads Immobilization technique (inactive cells)	Ethacridine lactate		Biosorption capacity was 26.76 mg/g and the removal efficiency was 89.93% for C_0_ = 60 mg/L; pH = 4; biosorbent dose = 2 g/L	[49]
Saccharomyces pastorianus residual biomass / calcium alginate composite beads Encapsulation technique (inactive cells)	Ethacridine lactate	pH = 2–10; initial concentration of the pharmaceutical compound = 20–60 mg/L; biosorbent beads dose = 1–3 g/L; time = 24 h; room temperature; batch system	Biosorption capacity was 21.39 mg/g and the removal efficiency was 85% for C_0_ = 50 mg/L; pH = 2; biosorbent dose = 2 g/L	[15]
Pandoraea sp. strain BT102 (bacterium-encapsulated in calcium alginate beads) (active cells)	p-Chloro-meta- xylenol	pH = 10 ± 0.2.; temperature = 37 °C; initial concentration of the pharmaceutical compound = 1–100 mg/L; biosorbent beads dose = 100 g/L of drug solution; time = 16 h; shaker at a rotating speed of 120 rpm; batch system	Maximum adsorption capacity was 961.7 mg/g for C_0_ = 100 mg/L after 4 h	[137]
**CHITOSAN—BASE FOR IMMOBILIZATION/ENCAPSULATION**				
	**Antibiotics**			
Saccharomyces cerevisiae/ chitosan composite beads (inactive cells)	Cephalexin	pH = 5–6; initial concentration of the pharmaceutical compound = 30–50 mg/L; biosorbent beads dose = 1 g/25 mL of drug solution; time = 12 h; room temperature; batch system	Maximum adsorption capacity was 22.78 mg/g for C_0_= 30 mg/L and pH = 5	[52]
	Rifampicin		Maximum adsorption capacity was 24.70 mg/g for C_0_= 50 mg/L and pH = 6	
Saccharomyces pastorianus/ chitosan composite beads (inactive cells)	Cephalexin	pH = 5–6; initial concentration of the pharmaceutical compound = 30–50 mg/L; biosorbent beads dose = 1 g/25 mL of drug solution; time = 12 h; room temperature; batch system	Maximum adsorption capacity was 28.42 mg/g for C_0_= 30 mg/L and pH = 5	[52]
	Rifampicin		Maximum adsorption capacity was 24.89 mg/g for C_0_= 50 mg/L and pH = 6	
**OTHER MATERIALS USED FOR IMMOBILIZATION/ENCAPSULATION**				
	**Anti-epileptics**			
Phanerochaete chrysosporium immobilized in wood chips (inactive cells)	Carbamazepine	pH =3.2 ÷ 4.5; temperature = 30 °C; the pharmaceutical compound added into the influent at 1.0 mg/L; time = 28 days; aerating mode; fixed-bed bioreactor	Removal efficiency on day 7, in coexistence with naproxen, was 61.30 ± 3.84% and biosorption capacity was 0.06 mg/g	[84]
	**Anti-inflammatories**			
Phanerochaete chrysosporium immobilized in wood chips (inactive cells)	Naproxen	pH =3.2 ÷ 4.5; temperature = 30 °C; the pharmaceutical compound added into the influent at 1.0 mg/L; time = 28 days; aerating mode; fixed-bed bioreactor	Removal efficiency on day 7 in coexistence with carbamazepine was 90.35 ± 4.37% and biosorption capacity was 0.17 mg/g	[84]

## Data Availability

Not applicable.
